# Rebuilding the Vascular Network: *In vivo* and *in vitro* Approaches

**DOI:** 10.3389/fcell.2021.639299

**Published:** 2021-04-21

**Authors:** Xiangfu Meng, Yunhui Xing, Jiawen Li, Cechuan Deng, Yifei Li, Xi Ren, Donghui Zhang

**Affiliations:** ^1^State Key Laboratory of Biocatalysis and Enzyme Engineering, School of Life Sciences, Hubei University, Wuhan, China; ^2^Department of Biomedical Engineering, Carnegie Mellon University, Pittsburgh, PA, United States; ^3^Key Laboratory of Birth Defects and Related Diseases of Women and Children of MOE, Department of Pediatrics, West China Second University Hospital, Sichuan University, Chengdu, China; ^4^Department of Obstetrics and Gynecology, West China Second University Hospital, Sichuan University, Chengdu, China

**Keywords:** vascular network formation, angiogenesis, cell therapy, decellularization, vessels on a chip

## Abstract

As the material transportation system of the human body, the vascular network carries the transportation of materials and nutrients. Currently, the construction of functional microvascular networks is an urgent requirement for the development of regenerative medicine and *in vitro* drug screening systems. How to construct organs with functional blood vessels is the focus and challenge of tissue engineering research. Here in this review article, we first introduced the basic characteristics of blood vessels in the body and the mechanism of angiogenesis *in vivo*, summarized the current methods of constructing tissue blood vessels *in vitro* and *in vivo*, and focused on comparing the functions, applications and advantages of constructing different types of vascular chips to generate blood vessels. Finally, the challenges and opportunities faced by the development of this field were discussed.

## Introduction

The purpose of tissue engineering is to exploit engineering methods to overcome physiological or medical difficulties. Its main objective is to facilitate tissue or organ transplantation and scientific research constructing functional tissue models *in vitro*. After a long period of research and development, the technology of tissue engineering has achieved encouraging results and milestones. However, a significant challenge currently remains concerning how to generate a large volume (>1 cm^3^) of viable, functional tissue. At present, the main difficulty in constructing larger tissues is the inability to form a vascularized network *in vitro* to supply oxygen and nutrition; thus, the central areas of fabricated tissues without vascularization tend to undergo rapid necrosis. Thus, tissue engineering research has focused on how to generate functional vascular networks. Herein, we will introduce the current progress of vascular network construction, including histological and cellular characteristics, using *in vivo* and *in vitro* strategies for angiogenesis, and the potential clinical applications. This review will first introduce the basic characteristics of blood vessels in the body and the mechanisms involved in angiogenesis of blood vessels *in vivo*. The current methods of constructing tissue vessels *in vitro* are also described within this review, including the use of vascular chips, and the functions, applications, and advantages of the vessels generated by these different methods are compared and analyzed.

## Structural Characteristics of the Vascular Network

### Histological and Cellular Fundamentals of Peripheral Vessels

The peripheral vascular system (PVS) includes all the blood vessels that exist outside the heart. The classification of the PVS is described below. Arteries have larger calibers and a better capability to bear high perfusion pressure. When arteries reach a specific organ, they branch into smaller vessels. As a result, there are two main types of arteries in the body: (1) elastic arteries and (2) muscular arteries. Elastic arteries consist mainly of large arteries and directly connect to the heart (such as the aorta and pulmonary arteries) and present more elastic features. Muscle arteries mainly distribute as medium-sized vessels, and their walls primarily contain smooth muscle.

The arterioles are the main vessels present in different organs and present relatively limited amounts of elastic tissue. Capillaries are thin-walled blood vessels composed of a single layer of endothelial cells. Based on the histological and cellular characteristics of capillaries, the exchange of nutrients and metabolites is achieved mainly through diffusion. Unlike arteries, venous pressure is very low. The venous wall is thin and less flexible. The veins receive blood from capillaries to complete the circulation system.

The function and structure of each segment of the PVS depends on the organ which it supplies, varying across different tissues. Aside from capillaries, most blood vessels are made of three layers. The adventitia, or outer layer, is mainly composed of loose connective tissue, which provides structural support and maintains normal vessel shapes. The tunica media, or a middle layer, is composed of elastic and muscular tissue, which determines the internal diameter of the vessel. The inner layer is formed by a single layer of endothelial cells, which are surrounding by elastic fibers.

### Vessel Formation: Morphological Changes and Signaling Cascades

There are several mechanisms involved in vessel formation and they are considered as different phases of the same biological process. Generally, vessel formation in an early development stage is defined as vasculogenesis, and describes the establishment of the first primitive vascular network. Next, angiogenesis follows the process of vasculogenesis, and consists of the formation of branches and vascular remodeling. Furthermore, angiogenesis is mainly used to describe vessel growth stemming from pre-existing ones, and represents the main vessel formation process in adulthood. Typically, vessel formation may be divided into three steps: (1) vasculogenic assembly, (2) vessel sprouting, and (3) vascular remodeling.

During embryogenesis, the formation of new blood vessels is a *de novo* process. Angioblasts (mesoderm-derived endothelial precursors) differentiate into a primitive vascular labyrinth (vasculogenesis) (Uhrin, 2019). Within this process, angioblasts differentiate into endothelial cells (ECs), and the ECs are recruited to form vascular cords (Baldwin, 1996). Next, the vascular cords are further specified into hierarchically differentiated vessels of arteries, capillaries, and veins. To enable this process, several molecular signaling mechanisms have been implicated in the initial steps of vasculogenic assembly. Notch signaling is highly expressed in arteries, but poorly expressed in veins (Conway et al., 2001; Gridley, 2010). Blocking Notch signaling contributes to the genesis of more veins and disruption of arteries, which indicates that Notch signaling pathway is a key molecular mechanism in the induction of arteriogenesis. Furthermore, ephrin components are associated with Notch signaling: EphB2 is a target of Notch within arterial ECs and binds to EphB4 of venous ECs. Vascular endothelial growth factor (VEGF) is also a crucial factor that triggers arterial differentiation of ECs, and is a downstream component of the Hedgehog pathway (Kim et al., 2008). Moreover, transcription factors are also expressed by specific ECs. FOXC1 and FOXC2 drive the expression of arterial gene signatures under the guidance of VEGF and Notch signals (Hayashi and Kume, 2008). While COUP-TFII has been identified as a driving factor of venous cell fate. In addition, according to the different pressure loading profiles of the arterial and venous system, mechanical loading also contributes to the differentiation of specific vessels. Finally, the arterio-venous segregation is driven by VEGF-C.

A second process of vessel formation is defined as sprouting angiogenesis: there are three changes that define blood vessel morphology, sprouting, bridging, and intussusception, which is facilitated by two main types of cells: the tip cells and stalk cells (Conway et al., 2001). Essentially, the process could be described as (1) tip-stalk cell selection; (2) tip cell navigation and stalk cell proliferation; (3) branching coordination; (4) stalk elongation, tip cell fusion, and lumen formation; and (5) perfusion and vessel maturation. At the onset of sprouting, the tip cells secrete matrix metalloproteases (MMPs) such as MT-MMP1 to degrade basement membrane. Plasminogen activator inhibitor-1 (PAI1) controls the amount of MMPs secreted to prevent over-degradation. Furthermore, the antiangiogenic molecules, angiostatin and endostatin, are also involved in this process and ensure the optimal sprouting direction. Additionally, detachment of mural cells is stimulated by angiopoietin-2 (ANG2) for tip cells movement in response to stimulation by VEGF. Next, VEGF/Notch signaling selects tip and stalk cells. Stalk cells express enriched Notch signaling and tip cells express low levels. Conversely, tip cells express higher levels of the Notch ligand DLL4. JAGGED1 (JAG1) is expressed in stalk cells and acts to inhibit DLL4 signal activity and maintain Notch activity. Filopodia guide tip cells by sensing attractive and repulsive cues. Filopodia formation is stimulated by CDC42 and by the endocytosis of EphB2/VEGFR2 receptors. ROBO4/UNC5B signaling promotes stabilization of the endothelial layer through inhibition of SRC. Notch-regulated ankyrin repeat protein (NRARP) and SIRT1 guide stalk cells stabilization. Both WNT and Notch pathways are also involved in stalk cell stabilization through interaction with NRARP and LEF1/ß-CATENIN. Finally, the contraction of the cytoskeleton of ECs generates the vessel lumen. Besides ECs themselves, a few other cell types also make significant contributions to the angiogenesis process through their interaction with ECs. For example, macrophage mediates the tip cells fusion and branch anastomosis, including VE-cadherin controlling cell adhesion (Potente et al., 2011; Hong and Tian, 2020). Furthermore, pericytes, vessel associated multi-functional support cells, promote endothelial sprouting during angiogenesis and provide guidance by spatially restricting VEGF signaling (Eilken et al., 2017). Pericytes can also penetrate tissues prior to EC ingrowth and create a path enabling subsequent effective angiogenic invasion by ECs (Ozerdem and Stallcup, 2003; Rajantie et al., 2004; Eilken et al., 2017).

The final phase is vascular remodeling, transitioning from a primitive towards a more stabilized and mature vascular plexus, involving steps such as adoption of a quiescent endothelial phalanx phenotype, basement membrane deposition, pericyte coverage, and branch regression. Stalk cells are regulated by blood flow shear stress-induced KLF2 signals. The up-regulation of KLF2 controls the remodeling of the vasculature. High KLF2 signaling ensures the endothelial quiescence cells form patent vessels, while low KFL activity induces ECs apoptosis and regression to terminate a vessel. The recruitment of pericytes is guided by platelet-derived growth factor receptor (PDGFR) ß, S1PR1, EphB2, and Notch3 signaling. The process of pericyte coverage and acquisition of an endothelial phalanx phenotype is the typical sign of vessel maturation. Further, hypoxic conditions trigger the expression of HIF-α, leading to the over-expression of sVEGFR1 and VE-cadherin to force neoangiogenesis so as to improve oxygen perfusion.

**FIGURE 1:**
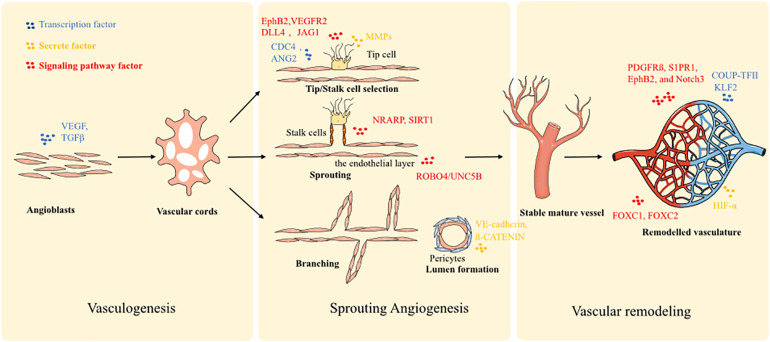
The major molecular regulators in vasculogenesis, angiogenesis, and vascular remodeling.

Given the extensive interplay of ECs, pericytes and macrophages at different stages along blood vessel formation, recapitulating the spatial and temporal interaction of this multi-cell-lineage system is of critical importance for enabling effective vasculogenesis and angiogenesis in tissue-engineered models. All the molecular biological processes were summarized in Figure 1.

## Inducing Vascular Regeneration *in vivo*

### Growth Factors Stimulating Angiogenesis

From an angiogenesis perspective, growth factors play critical roles in directing cells to initiate and maintain angiogenesis, since they are responsible for transmitting signals between cells and between cells and their microenvironment (Elaimy and Mercurio, 2018; Omorphos et al., 2021). Several growth factors have been widely studied and proven to be effective in promoting vascular regeneration, including VEGF, basic fibroblast growth factor (bFGF), placental growth factor (PLGF) and platelet-derived growth factor-BB (PDGF-BB) (Elaimy and Mercurio, 2018; Hao et al., 2018; Kant and Coulombe, 2018).

Despite the therapeutic potential of growth factors in promoting tissue regeneration, their clinical application is hindered by their limited stability and systemic side effects *in vivo*. The stability of growth factors can be enhanced by their incorporation into polymers to form a delivery system, which extends the *in vivo* half-life while maintaining bioactivity (Wang et al., 2017; Caballero Aguilar et al., 2019). In addition, delivery systems are able to provide spatial control of growth factor release, since they should remain at the target location long enough to induce a cellular response. After incorporation into hydrogel systems, growth factors, such as VEGF and bFGF showed improved ability to promote angiogenesis, long-term stability, and spatial localization *in vivo* compared to bolus injection or delivery in the aqueous solution of polymer (Adibfar et al., 2018; Shamloo et al., 2018).

### Delivery Strategies for Growth Factors

Three main methods have been applied for delivering angiogenic growth factors: (1) physical entrapment; (2) immobilization by covalent conjugation; and (3) the use of biocompatible micro/nano-particles. Physical entrapment can be achieved either by mixing growth factors with polymers before gelation or by physical absorption after polymer scaffold manufacturing (Tayalia and Mooney, 2009). By incubating with growth factors, such as VEGF, PDGF-BB, and transforming growth factor beta (TGF-β1), an alginate-sulfate hydrogel preparation triggered the formation of mature vascular structure (Freeman and Cohen, 2009; Sarker et al., 2019). Neovascularization responses may also be generated with hyaluronan hydrogel by mixing VEGF, bFGF, or KGF with polymers before gelation (Peattie et al., 2006; Pike et al., 2006; Graça et al., 2020). In order to achieve higher stability and prolonged release, growth factors can also be immobilized into polymers by covalent bonding. This usually requires chemical or enzymatic reactions between the growth factors and polymer scaffolds. For example, 1-ethyl-3-(3-dimethylaminopropyl)-carbodiimide hydrochloride (EDC) was used to covalently immobilize VEGF and angiopoietin-1 onto collagen scaffolds with controlled angiogenic bioactivities (Chiu and Radisic, 2010; Enriquez-Ochoa et al., 2020). In addition, growth factors can also be encapsulated or conjugated with biocompatible micro/nanoparticles, which allows precise control of the growth factor release rate and cellular uptake efficiency by fine-tuning the particle size and the surface-to-volume ratio (Tayalia and Mooney, 2009; Oduk et al., 2018). Likewise, the release of growth factors can be prolonged in the local microenvironment, leading to improved cellular responses and stability (Chen et al., 2010). It has been reported that following treatment with nanoparticle-encapsulated VEGF, vascularization and angiogenenic responses were achieved in the mouse hindlimb ischemia and rat myocardial infarction models (Golub et al., 2010; Oduk et al., 2018).

Since proteins generally lack stability *in vivo*, introducing growth factors by DNA transfer could provide sustained support for vascularization or angiogenesis. The delivery of VEGF gene using direct injection of naked plasmids or adeno-associated viruses (AAVs) has produced positive results in the activation of angiogenic genes and vascular regeneration, in critical limb ischemia and myocardial infarction models (Schwarz et al., 2000; Boden et al., 2016; Liu et al., 2017; Samatoshenkov et al., 2020). Endothelial progenitor cells (EPCs) with adenovirus transduction of the VEGF gene were tested in a mouse limb ischemia model and the implanted cells facilitated the neovascularization of the impaired region (Iwaguro et al., 2002). Similarly, adipose stromal cells are able to serve as a vector for growth factor delivery because of the angiogenic growth factors they secrete, and have demonstrated remarkable angiogenic potential in a limb ischemia model (Rehman et al., 2004; Zhao et al., 2017). However, with DNA transfer it is difficult to control the exact dosage, and risk of mutagenesis from genome integration is also an important issue that needs to be taken into consideration. Compared to DNA delivery, RNA is an attractive alternative because of its ability to induce an exogenous transient expression of growth factors without mutagenesis risk (Lui et al., 2014). The mouse myocardial infarction model was used to test the transplantation of modified RNA encoding VEGF by intramyocardial injection. Injection of the modified RNA increased capillary density and reduced the area of infarction while inducing the differentiation of epicardial progenitor cells towards endovascular cells (Zangi et al., 2013).

### Non-coding RNAs for Angiogenesis *in vivo*

Non-coding RNAs, which target mRNAs in a sequence-specific manner, contribute to the regulation of angiogenesis. miRNAs are a type of small endogenous non-coding RNA, which have been a focus of research for over a decade. A series of miRNAs have been shown to have pro- and anti-angiogenic characteristics. MiR-199a, miR-150, miR-145, and miR-153 interact with VEGF signaling, while miR-134, miR-153-3p, and miR-137 regulate Notch signaling activity, and are all involved in modulating angiogenesis (Zhao et al., 2020). MiR-320a and miR-27b have been administered in a preclinical study to re-establish neovascularization in retinopathy (Zampetaki et al., 2016). MiR-424 and miR-210 have been shown to have proangiogenic features in myocardial infarction (Hu et al., 2010), while miR-92 exerts antiangiogenic activity (Bonauer et al., 2009). Furthermore, several miRNAs (including miR-1, miR-133, and miR-126) have been used to inhibit angiogenesis as cancer therapy (Nohata et al., 2012).

In recent years, long non-coding (Lnc)-RNAs have also been discovered and their biological roles have been demonstrated more clearly. Lnc-SNHG1, H19, MIAT, ZFAS1, MEG8, MALAT1, NEAT1, and TUG1 have been identified for their ability to promote angiogenesis via targeting VEGF expression (Zhao et al., 2017; Hou et al., 2018; Long et al., 2018; Smith et al., 2018; Wang et al., 2018; Zhou et al., 2019; Barth et al., 2020; Li et al., 2020; Sui et al., 2020). Several *in vivo* delivery studies have confirmed their functional benefit in the prognosis of ischemic injury, especially of the heart and brain. Moreover, lnc-DIGIT, HIF1A, and XIST, targeting the Notch and DLL4 genes, modulate vascular network remodeling (Hayashi and Kume, 2008; Li et al., 2017; Miao et al., 2018; Tetzlaff and Fischer, 2018).

To deliver the non-coding RNAs *in vivo*, two common strategies are typically used: viral and non-viral introduction (Huang et al., 2020). For virus-based approaches, an AAV has been approved by the United States Federal Drug Administration for oligonucleotide delivery (Wang et al., 2019). Taking advantage of the AAV system, non-coding RNA inhibition and overexpression can be achieved by specific tissue infection based on different serotypes. Besides, adenovirus and lentivirus-based approaches have also been used to transfect animals in *in vivo* research studies. For the non-viral introduction, liposomes, nanoparticles, and hydrogels have been used to deliver non-coding RNAs for promoting vascular tissue regeneration (Li et al., 2018). Furthermore, chemical modifications of the oligonucleotide has been explored to further improve their *in vivo* stability and bioactivity, and include cholesterol modification, methoxyethyl modification, locked nucleic acid (LNA), and antagomirs strategies (Duygu et al., 2019).

## Cell Therapy *in vivo*

Besides biomolecular delivery, cell therapy is considered an alternative method for boosting angiogenesis. There are two main strategies for implanting cells to target tissues, one is the direct cell delivery without material support (Hur et al., 2004), and the other involves embedding cells into a specific biomaterial matrix (Blache and Ehrbar, 2018), such as hydrogel, to generate a compound construct. EPCs are considered to possess the most appropriate features suitable for neo-vascularization in ischemia, as they reside in the bone marrow and retain the ability for self-renewal and transformation into mature ECs (Ingram et al., 2005; Bogoslovsky et al., 2010; Raval and Losordo, 2013). Thus, this type of cell source has been applied in several preclinical studies of functional vascular regeneration, including peripheral ischemia, myocardial infraction (NCT02174939 and NCT03216733) and ischemic cerebral stroke (NCT01289795, NCT01468064, NCT02157896, NCT02605707, and NCT02980354). The studies by Jeevanantham et al. (2012) and Erbs et al. (2007) demonstrate positive results for cell therapy after myocardial infraction in promoting heart function and rebuilding coronal microvascular circulation. Although no evidence has been presented indicating that EPCs can differentiate into other cell types beyond ECs in the heart, in the nervous system, EPCs can integrate into the vascular endothelium in ischemic areas and stimulate neurogenesis (Bogoslovsky et al., 2010).

The three-dimensional (3D) matrix of hydrogel has desired permeability and can effectively enable the diffusion of nutrients and metabolic wastes to the embedded cells, thereby supporting their survival and physiological functions, and has been extensively used for cell encapsulation and delivery (Schmidt et al., 2008). Hydrogel materials have become valuable 3D scaffolds for vascular engineering since they share many features with the natural extracellular matrix (ECM), including high water content and viscoelastic properties. Many hydrogel materials also offers tunable degradability, and can be gradually replaced by the host’s ECM as the implanted tissues constructs mature *in vivo* (Langhans, 2018). Typically, the strategy includes four steps: (1) selection of cell sources: somatic cells, adult progenitor and stem cells, and pluripotent stem cells (PSCs) can be used to produce ECs; (2) establishment of hydrogel scaffolds: natural polymers, including collagen, poly(ethylene glycol) (PEG), gelatin methacrylate (GelMA), and synthetic polymers (including polylactic-co-glycolic acid) (PLGA), polycaprolactone (PCL), poly-L-lactic acid (PLLA), glycosaminoglycans (GAGs), hyaluronic acid (HA), and arginine-glycine-aspartic acid (RGD), have been used to manufacture hydrogel matrices for EC embedding and building micro-vessel systems (Khetan and Burdick, 2010; Chwalek et al., 2014; Blache and Ehrbar, 2018). (3) Compound functional graft building: the combination of scaffold, cells, growth factors, and mechanical stimuli recreates a functional microenvironment that stimulates tissue organization into an integrated engineered graft. (4) Graft implantation: the functional graft can be delivered in either in micro (10 μm) and nano (10 nm) molecules of gels for skin wound repair and osteogenesis in bone loss (Shen et al., 2016; Alarçin et al., 2018). Furthermore, injectable hydrogels can be used for intra-vascular delivery, especially in myocardial infarction (Yang et al., 2018).

## Engineered Recapitulation of the Vascular Circulation *In Vitro*

### Vascular Regeneration From Decellularized Matrix

The vasculature of native organs has complex spatial architecture that is essential for the organ-specific functions (Theocharis et al., 2016; Gilpin and Yang, 2017). The decellularized organ ECM also offers preserved essential ECM composition for supporting vessel formation, and serves as a reservoir for bioactive cues, such as growth factors and cell-adhesive mediators (Rhodes and Simons, 2007; Chan and Leong, 2008). Following decellularization, the porcine dermis showed retention of ECM proteins and expression of inherent growth factors, such as VEGF, TGFβ, and FGF, which have bioactive potential to stimulate vascular regeneration (Hoganson et al., 2010). The closed circulatory circuit within an organ is composed of multiple types of blood vessels with diverse functions, which are supported by the surrounding ECM (Thottappillil and Nair, 2015). Via decellularization, it is possible to preserve the composition and the overall architecture of specific blood vessel types and to achieve organ-specific vascular regeneration (Rhodes and Simons, 2007; Thottappillil and Nair, 2015). For example, sinusoids are capillaries that exhibit an incomplete basal membrane and widely exist in the adrenal glands, liver, spleen, and bone marrow (Augustin and Koh, 2017). In the decellularized human liver, the sinusoid structures are preserved and allow the migration of LX2 cells during recellularization (Mazza et al., 2015).

The ECM scaffolds used for regenerating large-diameter vascular conduit can be obtained from both decellularized vascular and non-vascular structures. The decellularized ureter and umbilical artery are capable of supporting endothelialization, leading to durable blood vessel formation in *in vivo* animal models (Narita et al., 2008; Gui et al., 2009). Recellularization for vascular scaffolds can be accomplished either *in vivo* by recruiting host’s cells following implantation or by *in vitro* culture with seeded cells (Yow et al., 2006). Following implantation, the decellularized allograft matrices demonstrated recellularization by recruiting host cells *in vivo* for ovine vessel and rat aortic conduit reconstructions (Ketchedjian et al., 2005; Assmann et al., 2013). The sources for *in vitro* recellularization can be mature vascular cells, such as ECs or smooth muscle cells, and EPCs (Zhang et al., 2007). For example, porcine iliac arteries can be decellularized and seeded with EPCs before transplantation, which allowed improved patency rates *in vivo* (Kaushal et al., 2001). However, efficient regeneration of complex microvascular structures, especially recapitulating their organotypic anatomy and functionality, remains challenging.

As an effort to address these challenges, advances in whole-organ perfusion decellularization in the past decade provide a convenient approach for producing organ-specific, acellular vascular bed allowing access to both macrovascular and microvascular compartments throughout the entire organ. For example, to rebuild the gas exchange function and attain vascularization, rat lungs were decellularized and seeded with ECs and epithelial cells or adipose-derived stromal cells within a bioreactor, which simulated a biomimetic culture conditions. Following orthotopic transplantation, the mature vascularized lung constructs was capable of providing gas exchange function *in vivo* (Ott et al., 2010; Doi et al., 2017). For the recellularization of a liver graft, hepatocytes and ECs were used to reconstruct the vasculature in the decellularized liver matrix (Uygun et al., 2010). Using perfusion recellularization, the embryonic stem cells or iPSC-derived ECs can be efficiently retained in the decellularized kidney scaffold, resulting in a uniform distribution in the vascular network and in glomerular capillaries, together with site-specific EC specialization (Bonandrini et al., 2014; Ciampi et al., 2019).

### Mimicking Angiogenesis *in vitro*

Compared to angiogenesis *in vivo*, models simulating angiogenesis *in vitro* can precisely control each component of vascular development, imitate the complex microenvironment and required factors in the body, with high repeatability. In current studies, *in vitro* vascular research usually uses differentiated ECs or adult EC lines and another type of cells (such as fibroblasts/mesenchymal cells/parenchymal cells, and even tumor cells) to be cultured under controlled 3D conditions. This creates a defined culture system for studying the effects of certain variables on angiogenesis. Two-dimensional (2D) *in vitro* models can be used to study the behavior of ECs, such as migration and proliferation; however, because 2D models lack the more physiological 3D environment, they cannot reflect the contribution of numerous typical characteristics of ECs, such as lumen formation and differentiation into tip and stalk cells. Therefore, constructing a 3D vascular model is particularly helpful for studying vascular tissue behavior with reproducible conditions of morphology and signals. Moreover, through reasonable engineering design, a high-throughput angiogenesis model has been developed, which facilitates screening of angiogenesis modulators.

Blood vessels are organs of luminal structure with essential transport and perfusion functions. Therefore, how to realize the perfusion and circulation function is a key task to accomplish for engineered vascular network *in vitro*. Tissue engineering methods for constructing 3D blood vessels *in vitro* mainly include self-organized cell sheets, 3D printed blood vessel networks and blood vessel chips (Sarker et al., 2018). In the self-organization approach, the growth and developmental process of the vascular system is stimulated, causing the EC to spontaneously form blood vessels in the hydrogel with native-like morphology and function. However, due to the randomness of self-organization, it remains difficult to perform fluid infusion. Although 3D printing blood vessels is convenient and automated, it is challenged by the difficulty to remove the hollow channels where the materials and blood vessel cells are fused, and the low spatial resolution.

Jeon et al. (2014) combined ECs with the ECM, and observed the generation of microvascular networks, in which ECs began to aggregate into a solid tube-like shape, with buds extending outward while responding to the effects of native-like growth factor combination of VEGF, TGFβ1, and Ang1. Belair et al. (2015) implanted iPSC-ECs and lung fibroblasts into fibrin gels within microfluidic devices. At the beginning of the culture, iPSC-ECs showed budding, migration, and invasion behavior, and were able to respond to fluid stimulation by extending in the direction of the fluid flow and forming perfusable lumens (Belair et al., 2015). van Duinen et al. (2017) developed a model of 96 individually addressable, 3D microvessels in a standardized microfluidic platform. It allows simultaneously analysis of the effects of different ECM/medium/cytokines (VEGF, TNFα, and IL-1β) on the barrier function of the engineered microvessels (van Duinen et al., 2017). The same group later refined this design and developed a more standardized high-throughput culture platform integrated into a 384-well plate, which formed 40 repeating units of three microfluidic channels. It can detect the effects of multiple factors and shows promise in application to screen vascular-related drugs (van Duinen et al., 2019). Kim et al. (2015) proposed a quantitative microfluidic angiogenesis screening platform (QMAS) that can monitor and quantify cell behavior from treatment with various drug concentrations, such as morphological changes, EC viability, and angiogenic bud formation. The platform has 14 connected chambers, with medium flowing through the gaps between each chamber to achieve a drug concentration gradient (Kim et al., 2015).

### Establishing Different Types of Microvessels on a Chip

At present, there are several different constructions and design strategies that have generated blood vessel chips. Generally, there are five types of vessel chips available as *in vitro* models: (1) microfluidic membrane: a microfluidic device incorporating a mechanically stretchable membrane that can capture the organ-level function of vascular and epithelial interfaces; (2) microfluidic scaffolds: a standalone microfluidic network that can be fabricated using a synthetic polymeric elastomer serving as a scaffold for the construction of vascularized functional tissues; (3) microfluidic hydrogels: consisting of hydrogels with built-in microchannels, that offer an alternative method to model tissue interfaces; (4) self-assembled microvasculature: which establish proper connections to microchannels —such a self-assembled microvasculature can be perfused with a microfluidic circuit; (5) open-well design: uses preformed parenchymal tissues, such as tumor spheroids, that can be inserted on a preformed vascular bed in a multistep seeding approach —the versatility of this platform is that it allows potential integration of the microvasculature with various models of dense solid tissues (Zhang et al., 2018).

Initial efforts focusing on building microfluidic chip devices with only vascular structures, primarily for validating the vascular functions in response to fluid flow. To date, several cell sources have been explored to generate microvascular networks, including colony-forming cell-derived ECs isolated from the cord blood, human dermal microvascular ECs, kidney peritubular microvascular ECs and HUVECs. These artificial tissues model the formation of a vascular barrier with perivascular interaction, vascular sprouting, a basement membrane, and ECM formation, and cytokine responses. Elevated gene expression of VCAM1, E-selectin, ICAM1, and leukocyte recruitment in response to TNF-a stimulation allows modeling of vascular dysfunction due to sepsis and inflammation. Furthermore, several molecular tests could be performed to evaluate the inhibitory effects on angiogenesis. Yasotharan et al. (2015) have built vessel chips using arteries from mice based on a microfluidic platform, and tested the calcium flux and dose-dependent vasoconstriction in response to phenylephrine, in the presence or absence of nifedipine.

**FIGURE 2:**
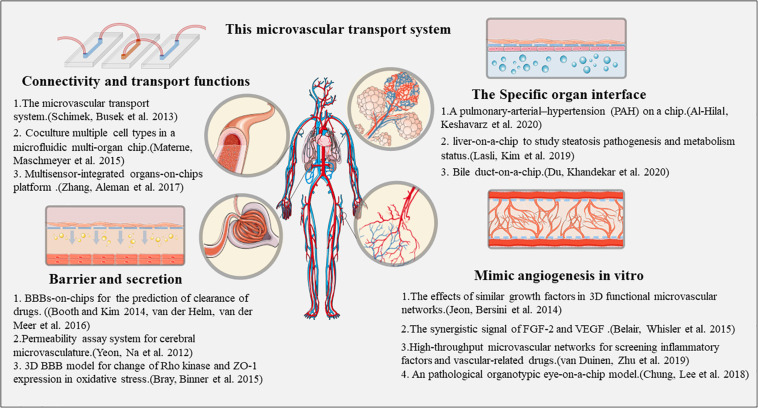
The major mimic strategies of vessels on a Chip.

Moving to engineering vascularized complex tissues, the blood-brain barrier (BBB) model has been generated by a hierarchical combination of ECs, neurons, and astrocytes based on a microfluidic platform (Andjelkovic et al., 2020). The sources of ECs for this purpose can be HUVECs, and primary brain-derived microvascular ECs. The BBB tissue models act as assays of drug activity, allowing evaluation of the flow, structure, and metabolic characteristics of brain vessels. Moreover, the incorporation of microvascular network is also important for studying tumor tissue biology as angiogenesis is essential for tumor growth. Thus, co-culture of various cancer cells with perfusable microvasculature allows generation of a virtual tumor tissue and provides a platform to observe tumor growth characteristics, screen drugs, and assess the efficacy of radiation therapy (Dijkstra et al., 2018). All the above information has been summarized in Figure 2.

## Practical and Clinical Applications of Vessels on a Chip

### Drug Testing

Preclinical drug screening is mainly performed using animal models. Although of great value, animal responses to drugs may show differences in terms of toxic doses and drug metabolism compared to human patients. Disease models tissue-engineered with patient cells can be used to accurately predict drug responses on tissue or even organ scales. Many current *in vitro* disease models often contain only the organ parenchymal cells without vascular support; thus, lack the ability to investigate the transport and metabolism of drugs in the vascular system (Fernandez et al., 2016). Compared with animal models and 2D experimental platforms, microvascular chips can conduct more detailed experiments on factors affecting vascular formation and have greater potential for use in vascular disease models to study pathological events and develop drug screening platforms. Besides, the microvascular chip systems have already made into commercial application beyond laboratory, focusing on high-throughput drug screening, precise diagnosis, disease modeling, and targeted therapy verification, which provide essential models for clinical management of cardiovascular diseases with opportunities of new discoveries (Portillo-Lara et al., 2019; Savoji et al., 2019).

In general, drug testing focuses on three aspects: (1) effects of drugs on vascular function. Microvascular networks and artery tissue models provide a platform to test drug effects on vessel growth and contraction, such as inhibitors of Rho kinase (Y27633), tyrosine phosphorylation (genistein), SRC kinase (PP2), and nitric oxide (L-NAME) (Ribas et al., 2016). (2) Molecules for blood barrier transportation. BBB and blood-retina barriers are always impediments for drug transportation and cellular translocation to particular tissues of interest to reach a proper therapeutic dose. Booth and Kim (2012) published their μBBB model, and demonstrated that co-culture with astrocytes resulted in an improved barrier functionality. Further, they showed that permeability coefficients in their model under dynamic conditions correlated well with *in vivo* brain/plasma ratios, demonstrating the potential of this model to predict drug clearance by the BBB (Booth and Kim, 2014; van der Helm et al., 2016). Cho et al. (2015) published a 3D BBB model, with the ability to record transmigration of neutrophils across the endothelial barrier and through the side channels, which was used to study ischemia by exposing the endothelium to low oxygen and low glucose levels and subsequently allowing reoxygenation to normal conditions. Formation of reactive oxygen species and the activation of Rho kinases as a result of oxidative stress was confirmed, as well as a decrease in tight junction ZO-1 expression. These *in vitro* culture models (van der Helm et al., 2016) have been used in combination with animal models as a supplementary testing platform, as at this stage the *in vitro* platforms alone are not yet suitable to reproduce all functional activities. (3) Chemical medication screening. Co-culture of cancer cells and the permeable microvascular network could induce tumor tissue growth *in vitro* (Bray et al., 2015), and offers the possibility for screening of chemotherapeutic agents. Drug screening has been performed to evaluate the inhibition efficacy on tumor growth and on the associated vasculature, by agents such as bortezomib, vincristine, mitomycin C, gemcitabine, vorinostat, tamoxifen, linifanib, axitinib, and sorafenib, pazopanib, oxaliplatin, folinic acid (leucovorin), and 5-fluorouracil (Zhang et al., 2018).

### Disease Modeling

Modeling a disease using patient-derived cells provides insight into the underlying pathology. Importantly, this approach provides huge advantages over the use of animal models, as animal models often cannot completely reproduce the physiological responses of human tissues (Cochrane et al., 2019).

An organotypic eye-on-a-chip model has been described, which mimics the retinal pigment epithelium (RPE)-choroid complex *in vitro*. This model consists of a RPE monolayer and adjacent permeable blood vessel network, which supports the barrier function of the outer blood-retina barrier (oBRB). The intact barrier function of the RPE-choroid complex is reconstituted while maintaining important structural features. Further, this model can successfully mimic the pathogenesis of the choroidal neovascularization (CNV). The alleviation of the pathological angiogenesis mechanisms can be modeled with bevacizumab, a clinical drug used in CNV treatment (Chung et al., 2018), utilizing blood outgrowth ECs (BOECs) as a disease-specific primary cell source to analyze vascular inflammation and thrombosis in vascular organ-chips or “vessel-chips” (Mathur et al., 2019). Similar concepts have been applied to other organ systems. Al-Hilal et al. (2020) reported a pulmonary-arterial-hypertension (PAH) model on a chip to elicit arterial remodeling under PAH. The authors fabricated a microfluidic device to emulate the luminal, intimal, medial, adventitial, and perivascular layers of a pulmonary artery. Three types of pulmonary arterial cells (PACs), endothelial, smooth muscle, and adventitial cells were seeded to form the model. Moreover, Lasli et al. used the human hepatocellular carcinoma (HepG2) cell line and HUVECs as spheroid cultures to establish a “liver-on-a-chip” model to study steatosis pathogenesis and metabolism status (Lasli et al., 2019). In addition, Du et al. (2020) attempted to build a “bile duct-on-a-chip” to achieve organ-level function as a specific vessel device to evaluate bile duct pathophysiological processes.

Moreover, disease models have also been built to study the blood vessels themselves by examining endothelial dysfunction. TNF-α stimulation has been used to explore the biological procedure of thrombus formation as a mechanical cue. Further, ECs binding of T cells and neutrophil extravasation has been used to study the activation of endothelial inflammation. However, there is no suitable model to evaluate the features of Kawasaki disease. Thus, vessels chips may provide an opportunity to approach the pathogenesis of coronary lesions of Kawasaki disease. Furthermore, this platform may contributes to understanding mechanisms that promote thrombus formation, plaque geometry, and the high permeability observed in the atherosclerotic endothelium.

### Tissue Regeneration and Repair

The limitations of organ transplantation due to insufficient organ supply have given rise to the development of regenerative medicine. Its main purpose is to replace missing or damaged tissues with materials and tissue-specific cells and their various combinations to promote structural and functional healing. Currently, only a few engineered tissues (skin, cartilage, and bladder) have reached clinical success, while biomaterials designed to replace more complex organs have not been commercialized because, unlike engineered skin, cartilage, or bladder tissue, cell viability and the optimal function of the construct cannot be maintained by diffusion alone, and oxygen and nutrients necessary for survival and organ integration cannot be effectively supplied following transplantation. Therefore, the generation of functional vasculature is critical to the clinical success of engineering and construction of organs (Bae et al., 2012).

The human body contains a rich blood vessel network, which is mainly used for supplying oxygen and nutrients. Therefore, for regenerative tissue culture models, a multi-organ chip system containing a blood vessel network chip is physiologically relevant. Schimek et al. (2013) established a human cardiovascular on a chip-based system, with the microvascular system co-cultured with skin and liver tissue, and demonstrated the ability of the microvascular chip to promote the survival of other tissues. Materne et al. (2015) proposed a protocol to co-culture multiple cell types in a microfluidic multi-organ chip, and successfully constructed a microenvironment of skin tissue and liver tissue and blood vessels, enabling the a circulation network. The development trend of organ chips is that of multi-organ combined chips; thus, researchers have attempted to integrate multiple types of chips into “body-chips.” Zhang et al. (2017) built an easy-to-use modular, sense-based, multi-organ platform, whereby multiple organoid models (human cardiac organoids and vascular organoid, and human liver organoids) are connected by micro-organ chip technology, which can systematically study and analyze the ability of multiple organ interactions, with the advantages of continuous, dynamic, and real-time monitoring (Zhang et al., 2017).

Engineering a functional alveolar-capillary interface (lung), large functional myocardial tissue with suppling vessels tissue (heart), functional hepatic tissue combining hepatocytes and the bile duct (liver), functional renal tissue comprising permeability and filtering features (kidney) is the main goal for organ tissue construction from a regenerative perspective. Furthermore, mimicking the 3D arterial architecture, vascularized skin would also be more helpful for wound repair. Thus, the combination of functional artificial tissue with a vascularized network is a critical, desired objective for regenerative medicine. The establishment of blood vessels in different organs and different types of “organ on the chip” is summarized in Table 1.

**TABLE 1:** Build the blood vessels in different organ and different type of “organ on the chip.”

Chip type	Cell types	Hydrogel	Features	Reference
Angiogenic high-throughput platform	HUVEC-VeraVec^TM^ human endothelial cells	4.0 mg/ml solution of collagen type I	High-throughput, perfusion rocker; optimize the combination of angiogenic factors	van Duinen et al., 2019
Angiogenic chip	Human umbilical vein endothelial cells or RFP-expressing HUVECs	2.5 mg/ml solution of type I collagen	Screen for anti-angiogenic therapeutic drugs	Kim et al., 2015
Blood–brain barrier chip (BBB chips)	The brain endothelial cell line bEnd.3 and the glial cell line C6	Collagen IV/fibronectin	Quantified trans-endothelial electrical resistance (TEER); the permeability of the blood-brain barrier was measured	van der Helm et al., 2016
Cerebral vasculatures chip	Primary cultured HUVECs and human astrocytes	Attachment	Testing for chip based permeability measurement of drugs	Yeon et al., 2012
Tumor angiogenesis chip	HUVECs and MSCs, cancer cell line	starPEG solution	Mimic tumor angiogenesis microenvironments *in vivo*	Bray et al., 2015
Wet-AMD on a Chip	HUVECs and normal human lung fibroblasts, ARPE-19 cell line	fibrinogen solution	Modeling the pathogenesis of CNV especially in terms of morphogenesis	Chung et al., 2018
Vessel-chips	Endothelial progenitor cells from blood	Type I rat-tail collagen	Modeling vascular inflammation and thrombosis	Mathur et al., 2019
Pulmonary-arterial-hypertension (PAH)-on-a-chip	ECs, SMCs, and ADCs isolated from healthy human and patients with idiopathic PAH (IPAH)	Type I collagen solution	Modeling the PAH pathophysiology on the device	Al-Hilal et al., 2020
Liver-on-a-chip	HepG2 (from ATCC HB-8065) and HUVECs (from ATCC PCS100-010)	Spheroid in suspension	Modeling the nonalcoholic fatty liver disease	Lasli et al., 2019
Microvascular transport system	Human dermal microvascular endothelial cells	Attachment	Modeling the transport function of the human cardiovascular system	Schimek et al., 2013

## Discussion and Further Challenges

Although rapid progress has been made in the field of vascular tissue engineering in the past decade, the lack of functional vasculature has been widely regarded as one of the main obstacles hindering the effective reproduction of *in vivo* physiology. So far, the method of vascular reconstruction using acellular matrix in the body is mainly aimed at the reconstruction of large blood vessels. The real perfusion function of the whole blood vessel network can only be achieved following transplantation into the body and anastomosis with the host blood vessels. Chips have many recognized advantages as a convenient *in vitro* model of organ function and disease. Therefore, *in vitro* engineered pre-vascularization followed by embedding other organ models into the pre-established capillary bed, can be regarded as a way to realize the co-cultivation of microvascular chips and other organs.

### Bottleneck of Building Tissue-Engineered Blood Vessels

Building a functional vascular network has long been recognized as a major challenge in tissue engineering. Although we have now developed various types of vascular chips and printed vascular networks, there remains a lack of effective methods for regulating vascular network conformation and vascular scale, as well as a lack of commercially available models that are capable of supporting physiological perfusion and implantation. There is an unmet need to identify proper sources of patient-derived cells to be applied in organ-on-chip models to increase our mechanistic understanding of diseases and to assess the efficacy of drugs. The interactions between blood flow, vessel architecture, cells, and parenchymal tissues vary drastically from organ to organ. These differences largely arise from the structural, compositional and functional differences of the vasculature in different organs. The organ-specific origin of endothelial cells can have a profound effect on the biological activity. Many commonly used generic ECs, such as HUVECs, lack the characteristics presented by microvascular endothelial cells, which is an important feature for model systems. Thus, the choice of ECs will depend on the specific organ model and the corresponding parenchymal tissues. In the method of using acellular matrix and whole organ perfusion to build vascular network, the cell sources are always limited within a few cellular types, which is probably insufficient to establish functional, organotypic circulation network. Advances in targeted differentiation of stem cells and primary tissue isolation techniques will help us develop more organ-specific ECs (Lin et al., 2019). Taking advantage of these innovations, we expect continuing progresses toward engineering organ-specific vascular systems for improved understanding of the physiological and molecular mechanisms of organ and tissue formation.

The application of acellular matrix for rebuilding vascular network is superior compared to other synthetic matrices, but it remains difficult to fully recapitulate the native growth factor environment. The material used for the blood vessel chip should also consider the characters of material to support cellular growth in tunnel formation. Besides natural scaffolds, polydimethylsiloxane (PDMS) is the one of most widely used synthetic materials, which demonstrates the features of insulation, non-conductivity, resistance to leakage, good biocompatibility, easy oxygen permeability, and good optical properties. However, PDMS is difficult to be used for regeneration and transplantation applications due to its non-degradability. With the development of new materials, acellular matrices, and artificial polymers should be designed in combination with each other to facilitate the building of functional vascular network (Zhang et al., 2018).

Materials for construction of chips, cellular fidelity, multiplexing, fluid handling, scalable production, and validation of organ-on-a-chip devices are all areas requiring further study (Zhang et al., 2018). Material modification techniques should be developed to enable the modulation of material biocompatibility as well as their function on proliferation and differentiation. In addition, materials to be in contact with blood, there has always been a challenge of potential thrombosis, thus material modifications that reduce thrombogenicity is an area of extensive research for future development of vascular interfaces (Greco Song et al., 2018). In addition, computational modeling that enables the design of physiologic network architecture, including channel size, hierarchical structure, flow rate control are is desired (Chandra and Atala, 2019).

### Cross-Cooperation Across Research Fields

To build vascular network mentioned in this article is a field that requires interdisciplinary methods, knowledge, and technologies in biotechnology, tissue engineering, vascular biology, biomaterials, cell engineering, and stem cell biology (Li et al., 2014). In order to achieve effective and functional vascularization of biological materials, it is necessary to make the combination and synchronization of several factors that effectively characterize tissue vascularization *in vitro*, incorporating the inherent characteristics of biological materials and adapting to scaffolds. The biological concerns include how to control vascular stimulation by pro-angiogenic molecules, appropriate culture conditions, and fluid shear stressor. When these factors are integrated on the chip, they may affect the growth and function of blood vessels (Bramfeldt et al., 2010; Zhang et al., 2018).

#### Large or Small Vessels

At present, the application of large-caliber stent vessels in clinical applications will allow good blood flow perfusion and connectivity in the short-term, although in the long-term, further embolic events will often occur due to the deposition of blood components. The function, and often the transplantation of such large-caliber blood vessels, is limited to less than 1.5 cm in length, which is relatively costly (Fukunishi et al., 2016). The ideal vascular graft should be able to undergo rapid vascular remodeling by promoting host cell infiltration and encourage accompanying stent degradation. The perfusion function of small-caliber blood vessels and the maintenance of fluids is much more difficult to achieve. Therefore, to reconstruct large scale blood vessels, the acellular matrix-based angiogenesis is considered as optimal method with low rate of thrombosis occurrence and well anastomosis. Compared with the vascular chip, it can be used as a more convenient technology to verify or reproduce the function of part of the blood vessels in the body to simulate vascular-related diseases. In addition, due to the advantages of the repeatability and easy expansion of the artificial vascular chip, it offers the promise to enable commercial applications in high-throughput drug screening.

## Author Contributions

DZ, YL, and XR conceived of the presented idea, supervised the project, and contributed equally to the final version of the manuscript. XM, YX, and JL summarized the reference and draft the manuscript. CD helped with collected the reference. XM draft the table. DZ organized the figure with online free material. All authors contributed to the article and approved the submitted version.

## Conflict of Interest

The authors declare that the research was conducted in the absence of any commercial or financial relationships that could be construed as a potential conflict of interest.
